# Characterization of the Anti-Influenza Activity of the Chinese Herbal Plant *Paeonia lactiflora*

**DOI:** 10.3390/v6041861

**Published:** 2014-04-23

**Authors:** Jin-Yuan Ho, Hui-Wen Chang, Chwan-Fwu Lin, Chien-Jou Liu, Chung-Fan Hsieh, Jim-Tong Horng

**Affiliations:** 1 Department of Biochemistry, Chang Gung University, Kweishan, Taoyuan 333, Taiwan; E-Mails: jinyuanho@gmail.com (J.-Y.H.); a86x84@hotmail.com (H.-W.C.); b8921043@gmail.com (C.-J.L.); doctordoctorkk@yahoo.com.tw (C.-F.H.); 2 Department of Cosmetic Science, Chang Gung University of Science and Technology, Kweishan, Taoyuan 333, Taiwan; E-Mail: cflin@gw.cgust.edu.tw; 3 Research Center for Emerging Viral Infections, Chang Gung University, Kweishan, Taoyuan 333, Taiwan; 4 Department of Medical Research, Chang Gung Memorial Hospital, Kweishan, Taoyuan 333, Taiwan

**Keywords:** antiviral, Bai Shao, herbaceous peony, influenza virus, *Paeonia lactiflora* Pall

## Abstract

Bai Shao (BS, the root of *Paeonia lactiflora* Pall.), a common Chinese herb in many recipes used to treat viral infection and liver diseases, is recognized for its ability to nourish menstruation, its Yin convergence, and as an antiperspirant. However, the mechanism and components for its antiviral function remain to be elucidated. In this study, an ethanolic extract of BS was further partitioned into aqueous and organic parts (EAex) for *in vitro* functional study and *in vivo* efficacy testing. EAex exhibited an IC_50_ of 0.016 ± 0.005 mg/mL against influenza virus A/WSN/33 (H1N1), with broad-spectrum inhibitory activity against different strains of human influenza A viruses, including clinical oseltamivir-resistant isolates and an H1N1pdm strain. The synthesis of both viral RNA and protein was profoundly inhibited when the cells were treated with EAex. A time-of-addition assay demonstrated that EAex exerted its antiviral activity at various stages of the virus replication cycle. We addressed its antiviral activity at virus entry and demonstrated that EAex inhibits viral hemagglutination and viral binding to and penetration into host cells. *In vivo* animal testing showed that 200 mg/kg/d of EAex offered significant protection against viral infection. We conclude that BS possesses antiviral activity and has the potential for development as an anti-influenza agent.

## 1. Introduction

Influenza is a respiratory infection caused by the influenza virus, which is transmitted mainly through airborne aerosols of respiratory secretions and direct or indirect contact with infected people or their belongings. Patients may have coughing, fever, and a running nose. In some severe cases, influenza may cause death. Influenza has caused several epidemics or pandemics, including the 1918 Spanish (H1N1), the 1957 Asian (H2N2), the 1968 Hong Kong (H3N2), and the 2009 Mexican pandemics (H1N1pdm) [1], due to its high mutation rate and its ability to cause cross-species infections. The H5N1 [2] and H7N9 [3] strains have spread within birds, but have lately become a potential threat to humans because of antigenic drift and gene reassortment [4].

Influenza A virus belongs to the Orthomyxoviridae family, and contains eight negatively stranded RNA segments, which encode at least 12 proteins, including the RNA-dependent RNA polymerase complex (RdRp): PA, PB1, PB2, and NP, the outer-membrane proteins: M2, HA, and NA. HA and NA are the most abundant proteins on the viral surface [5]. The serotype of influenza A virus is determined by HA (H1 to H17) and NA (N1 to N10) [6]. HA interacts with sialic acid for virus entry [7,8], and NA assists progeny virus release by cleaving sialic acid on the cell membrane [9]. However, NA has recently also been shown to facilitate virus entry [10,11].

After virus entry, virus particles fuse with the endosome, the M2 ion channel allows protons to enter the virus, changing its pH environment, the virus structure is disrupted, and ultimately the viral genome is released into cytosol, where transcription and translation take place [12]. Currently, there are two classes of anti-influenza drugs, M2 and NA inhibitors. Amantadine and rimantadine are inhibitors of the M2 ion channel [13,14], which impedes the release of virus genome into the host. Oseltamivir and zanamivir (Relenza) are NA inhibitors that block NA from hydrolyzing the binding of host neuraminic acid and HA, thus preventing the virus from spreading. However, there has been an increasing number of cases of virus resistance being reported [15]. The rise of resistant viruses has become a serious problem, although several groups have demonstrated that a combination of oseltamivir and ribavirin treatment has reduced the death rate resulting from H5N1 infection in a mouse model of influenza [16]. Combined treatment with amantadine, ribavirin, and oseltamivir has more significant synergistic effects than any combination of just two of these drugs [17]. In addition to combinations of available drugs, the development of new drugs is desperately needed.

Our group has characterized the anti-influenza virus activities of two decoctions, Ko-Ken Tang (KKT) [18] and Ma-Xing-Shi-Gan-Tang (MXSGT) [19]. KKT inhibits virus replication by inhibiting the PI3K/AKT signaling pathway and viral RNP nuclear export, where MXSGT inhibits virus entry. An extract of Bai-shao (BS) has been proven to possess antibacterial [20], anti-HBV [21], and anti-HSV activities [22]. In this study, we characterized its anti-influenza activity and demonstrated its *in vivo* efficacy using an animal model of influenza.

## 2. Results and Discussion

### 2.1. Antiviral Activity of BS Ethylacetate Extracted Fraction (EAex) in MDCK Cells

Ethanol-extracted BS was partitioned to EA-soluble (EAex) and aqueous fractions for antiviral tests. The neutralization and cytotoxic assays suggested that EAex contained unknown substances that inhibited influenza virus WSN (H1N1) replication (IC_50_: 0.016 ± 0.005 mg/mL by anti-CPE neutralization assay, and 0.015 ± 0.001 mg/mL by plaque reduction assay) and had little cytotoxicity (CC_50_: 0.210 ± 0.022 mg/mL) (Table 1). However, there was barely any antiviral activity in the aqueous solution (data not shown). We expanded the scope of our study to test its antiviral activity on different strains of influenza A (flu A) viruses, influenza B (flu B) viruses, and enteroviruses (Table 1). It is noteworthy that EAex is capable of inhibiting several flu A, including two Tamiflu-resistant viruses (A/TW/6663/09 and A/TW/7717/09) and two H1N1pdm viruses (A/TW/206/09 and A/TW/167/09). In addition to that, EAex also inhibits several H3N2 flu A viruses and two flu B viruses (B/TW/70325/05 and B/TW/99/07). However, EAex did not inhibit enteroviruses (EV71/Tainan/4643/98, Echovirus 9, and Coxsackievirus A 16). Together, these results indicate that EAex exhibits a broad inhibitory spectrum toward H1N1 and H3N2.

### 2.2. Inhibitory Mechanism Determined by a Time-of-Addition Assay

**Figure 1:**
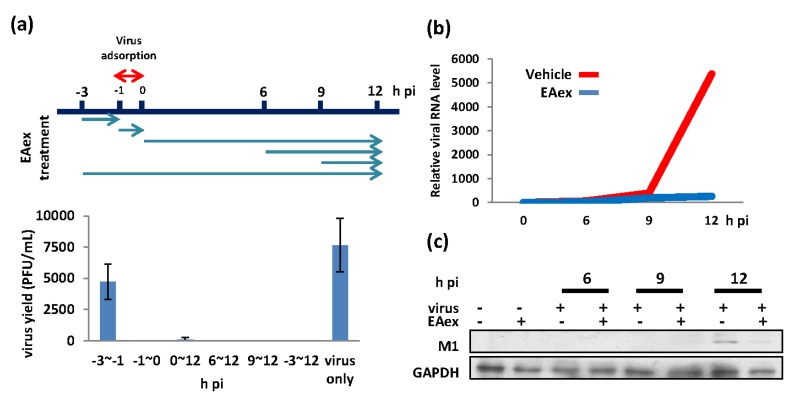
Determining the mode of action of EAex by (**a**) time-of-addition assay, and inhibition of (**b**) viral RNA and (**c**) protein production. MDCK cells were infected with influenza A/WSN/33 (MOI: 0.01) with 0.025 mg/mL of EAex added at the indicated time points. For RNA and protein experiments, 0.025 mg/mL of EAex was added after virus absorption. Viral M1 was normalized with GAPDH in both (**b**) RNA and (**c**) protein level, with or without EAex.

To understand the mechanism of EAex against flu A, we performed a time-of-addition assay to examine at which stage EAex inhibits flu A replication within a single life cycle. As shown in Figure 1a, EAex exerted its antiviral activity at almost all stages during virus replication, except the preadsorption stage (−3 to −1 h pi (post infection) (Figure 1a) with less inhibitory effect. We then examined the viral RNA (Figure 1b) and protein expression (Figure 1c). Our results showed that EAex inhibited viral RNA replication significantly (over 10-fold inhibition), but with a minor inhibitory effect on the relative period of protein level (from 1.0- to 1.4-fold), suggesting that the EAex-mediated inhibition of viral titer was a result of reduced viral RNA synthesis.

**Table 1:** Inhibition spectra of EAex against clinical isolated influenza virus and oseltamivir-resistant viruses.

	Concentration (mg/mL)		
	CC_50_^a^	IC_50_^b^	SI ^c^
**Cytotoxic effect**			
MDCK	0.210 ± 0.022		
RD	0.029 ± 0.001		
**Influenza viruses**			
A/WSN/33 (H1N1)		0.016 ± 0.005 ^b^	13.5
		0.015 ± 0.001 ^d^	14
A/TW/6663/09 (H1N1) *		0.005 ± 0.001	42
A/TW/7717/09 (H1N1) *		0.005 ± 0.001	42
A/TW/206/09 (H1N1)		0.007 ± 0.001	23
A/TW/167/09 (H1N1)		0.005 ± 0.001	42
A/udorn/72 (H3N2)		0.050 ± 0.001	4.2
A/3446/02 (H3N2)		0.003 ± 0.0004	70
A/TW/2289/12 (H3N2)		0.017 ± 0.008 ^d^	12.4
A/TW/3003/12 (H3N2)		0.026 ± 0.006 ^d^	8.1
B/TW/70325/05		0.036 ± 0.004	5.8
B/TW/99/07		0.042 ± 0.015	5
**Enteroviruses**			
EV71/Tainan/4643/98		>1	
Echovirus 9		>1	
Coxsackievirus A16		>1	

MDCK or RD cells were infected with influenza virus or enterovirus, respectively, and treated with a serial dilution of EAex. The values are mean ± SD of the results of two to three independent experiments. ^a^ CC_50_ was determined with an MTT assay. ^b^ IC_50_ was determined with an anti-CPE (neutralization) assay. ^c^ SI (selectivity index) is the ratio of CC_50_ to IC_50_; ^d^ IC_50_ was determined by plaque reduction assay. * Clinical Tamiflu-resistant strains [23].

### 2.3. EAex Inhibits the PI3K-AKT Pathway

**Figure 2:**
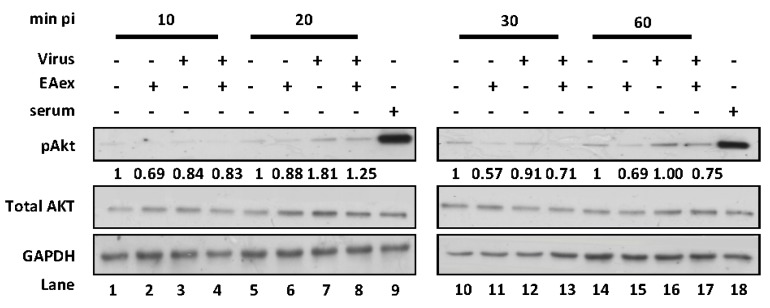
EAex inhibited the influenza virus-induced pAKT pathway. Influenza A/WSN/33 (MOI: 0.1) was incubated with 0.025 mg/mL on ice for 1 h. The mixture was then used to infect A549 cells, which had been incubated overnight in serum-free medium. Cell lysates were harvested at 10, 20, 30, and 60 min after virus addition, and GAPDH, AKT, and pAKT (S473) were detected by immunoblotting. P: positive control; A549 cells were incubated in serum-free MEM for 24 h before treatment with 2% FBS in MEM for 1 h. These figures are representative of three independent, reproducible experiments. Relative pAKT expression was normalized with AKT, and then normalized to the mock infection at each time point.

From a previous study, it is known that the influenza A virus induces PI3K/AKT pathways during virus replication, and then the S473 of AKT is phosphorylated [24]. Another study suggested that inhibition of PI3K activity at the early stage of virus infection reduces progeny virus production [25]. Moreover, viral protein synthesis is reduced by suppression of AKT phosphorylation [18,26]. Thus, we examined whether EAex inhibits virus-induced PI3K/AKT pathway activation. In a time-course assay, virus-induced pAKT was increased at 20 min (Figure 2, lane 7), but the pAKT was decreased compared with the virus control (lane 8) in the presence of EAex, demonstrating that EAex might inhibit viral-induced AKT phosphorylation at an early stage of viral infection.

### 2.4. EAex Inhibits Viral Entry

**Figure 3:**
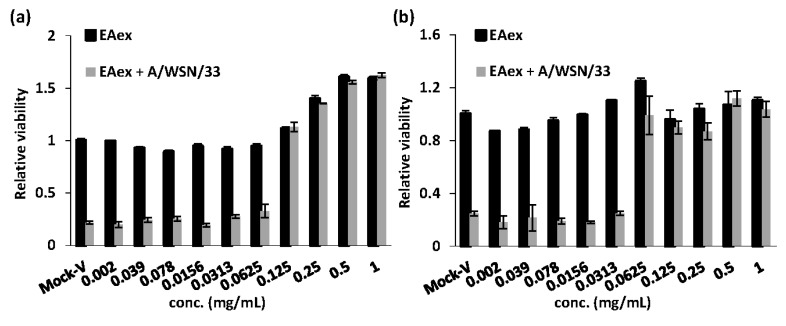
Inhibition of viral entry in attachment and penetration assays. (**a**) Before infection, MDCK cells were chilled on ice for 20 min. The cells were infected with 3TCID_50_ of influenza virus A/WSN/33 and treated with different concentrations of EAex on ice for 60 min. The medium containing unbound virus was removed, fresh FBS-free medium was added, and the cells were incubated for 72 h. Cell viability was measured using an MTT assay. Data are presented as the means ± SD of three independent experiments; (**b**) MDCK cells were prechilled for 20 min and infected with 3TCID_50_ of influenza virus A/WSN/33 on ice for 30 min. After the unbound virus was removed, the infected cells were supplied with FBS-free medium containing various concentrations of EAex and incubated at 37 °C for 1 h. Finally, the viruses that had not penetrated the cells were inactivated with HBSS (pH 2) and then neutralized with HBSS (pH 11). Cell viability was measured using an MTT assay. Data are presented as the means ± SD of three independent experiments.

According to the results of a time-of-addition assay and pAKT results, EAex inhibited virus replication at an early stage (Figure 1a and Figure 2). Thus, we further investigated whether EAex inhibits virus attachment to or entry into cells using attachment- and penetration-inhibition assays. In the attachment-inhibition assay, virus and serially diluted drugs were coincubated on ice during an absorption step; the amount of attached virus was determined by its virulence on cell viability, which was determined by an MTT assay. As shown in Figure 3a, inhibition of viral attachment was dose dependent and the IC_50_ of EAex inhibiting virus attachment was 0.058 ± 0.013 mg/mL against A/WSN/33. Two additional strains, A/TW/7717/09 and B/99/07, showed similar IC_50_ levels of 0.057 ± 0.008 and 0.074 ± 0.001 mg/mL, respectively (Supplementary Materials Figure S1b). We continued to examine whether EAex inhibits virus entry into cells using a penetration assay. Unlike in the attachment assay, virus was preadsorbed to cells on ice for half an hour, then the unbounded virus was washed away, and the cells incubated with serially diluted EAex at 37 °C to determine virus entry into the cells. Subsequently, cells were washed again to remove surface virus contamination, and cell mortality caused by penetrated viruses was determined by MTT assay after three days incubation. As shown in Figure 3b, the penetration IC_50_ of EAex was 0.032 ± 0.014 mg/mL against A/WSN/33, and the relative IC_50_ against A/TW/7717/09 and B/99/07 were 0.036 ± 0.005 and 0.020 ± 0.004, respectively (Supplementary Materials Figures S2a,b). These results suggest that EAex is capable of inhibiting virus entry at both attachment to and penetration into host cells.

### 2.5. Effect of EAex on HA and NA Activity

**Figure 4:**
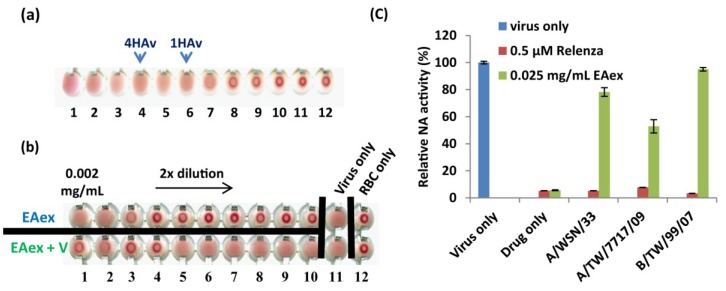
Effect of EAex on the HA and NA activities of influenza virus A/WSN/33. (**a**) Two-fold diluted WSN was incubated with RBCs of guinea pigs to determine the virus titer (1 × HAv); (**b**) RBC were incubated with influenza virus A/WSN/33 at 4 × HAv on ice for 1 h in medium containing different concentrations of EAex. The controls were RBCs mixed with PBS and EAex. EAex was serially diluted two-fold from 2 µg/mL to 4 ng/mL, shown from left to right; (**c**) Influenza virus A/WSN/33 was incubated with EAex or zanamivir for 30 min. MU-NANA (60 µM) was added and the reaction was incubated at 37 °C for a further 1 h. Stop solution was used to terminate the reaction and the fluorescence was measured.

To clarify whether EAex inhibits virus entry by inhibiting viral proteins, HA and NA, we performed HAI and NA activity assays. We utilized the signature effect of HA, a phenomenon called “hemagglutination,” which prevents red blood cell (RBC) from clotting in the presence of HA (Figure 4a) [19]. A two-fold serial dilution of EAex from 2 µg/mL (Figure 4b, lane 1) to 4 ng/mL (lane 10) was added with RBC and virus. As expected, virus *per se* caused hemagglutination (lane 11), and no hemagglutination for EAex alone starting from 500 ng/mL (lane 3, upper row), while EAex caused hemolysis at higher concentrations (lanes 1 and 2, upper row). EAex inhibited virus-induced hemagglutination at a lowest concentration of 250 ng/mL (lane 4, lower row), suggesting that EAex may exert part of its antiviral function through inhibiting HA–sialic acid interaction. In addition to HAI assay, we also examined whether EAex inhibits NA activity. As shown in Figure 4c, NA activities of two flu A strains were inhibited at around 20%–40% (lanes 3 and 4), but there was no inhibition of the flu B virus (lane 5). These results suggest that EAex inhibits both HA and NA for viral entry [10,11].

### 2.6. In Vivo Efficacy Test Using a Mouse Model of Influenza

**Figure 5:**
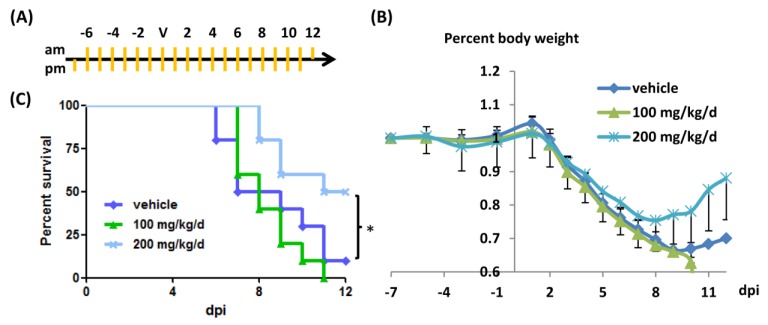
Efficacy of EAex on mouse models. EAex or Tamiflu were prepared in 5% DMSO, and mice were administered with 100 µL of solution twice a day by *per os* (daily uptake was as indicated). (**a**) indication of administration (orange lines) and virus challenge (V); (**b**) average body weight percent (normalized with D-7 body weight); (**c**) Survival rate comparison of DMSO and EAex (100 mg/kg/d or 200 mg/kg/d).

The results described above show that EAex could inhibit virus replication effectively. Thus, we further validated the antiviral efficacy of EAex using a mouse model. Initially, mice were administered EAex one day before viral challenge, and the body weight and survival rate were recorded every day. We found that there were some protective effects of EAex, but we also noticed that EAex alone caused mice to show slight fur ruffling on the first few days of observation (data not shown). Despite that, mice treated with EAex recovered faster than mice in the group treated with DMSO, although there was no significant difference in their survival rate. Thus, we prolonged drug administration for a further week before virus challenge, and then monitored the effects of EAex against influenza virus (Figure 5a). As shown in Figure 5b, administration of EAex (200 mg/kg/d, twice a day) increased the rate of body weight gain during the recovery phase. Moreover, it also significantly protected mice from death (Figure 5c; increase in survival rate of 40%).

### 2.7. Discussion

The use of traditional Chinese herbal medicine has been accepted in many countries. In this study, we demonstrated that BS possesses the anti-influenza virus activities both in cell culture and in our mouse model of influenza. The spectrum of inhibition showed that EAex inhibits a broad spectrum of influenza viruses, including H1N1, H3N2, and oseltamivir-resistant strains.

Our time-of-addition assay suggested that EAex inhibits viral RNA replication and protein translation (Figure 1b,c). Therefore, we asked whether reduced viral RNA and protein levels resulted from inhibition of RNP complex. As shown in Supplementary Material Figure S3, EAex did not suppress RNP complex activity at either 24 or 48 h. We also examined M1, NP, and HA distribution with time. As shown in Supplementary Material Figures S4, protein distribution patterns were not changed in the presence of EAex. Thus, how EAex inhibits influenza virus after the virus enters host cells at an early stage of infection remains incompletely understood.

Our results have proven that EAex can effectively inhibit virus propagation over the entire virus life cycle, especially in virus entry and release. As EAex is a mixture, we would like to examine whether any known major components of BS possess the antiviral properties. According to a previous report, six compounds are present in the crude organic extract of BS, namely gallic acid (anti-HIV and anti-HSV effects [27], and anti-human rhinovirus effects [28]), oxypaeoniflorin, albiflorin, paeoniflorin, benzoic acid, and paeonol, which are antioxidants and may possess antiviral properties. We examined three commercially available compounds (gallic acid, albiflorin, and paeoniflorin), but none of them inhibited virus replication effectively (data not shown). This suggests that unknown substances within EAex are capable of inhibiting influenza virus infection.

Our results provide clear evidence regarding how BS EAex inhibits influenza virus A replication. EAex primarily exerts antiviral activity by (1) inhibiting the PI3K/AKT pathway (Figure 2); (2) inhibiting virus attachment (Figure 3a) and virus entry (Figure 3b); and (3) inhibiting HA (Figure 4b) and NA activity (Figure 4c). Although in this study we focused on the inhibition of virus replication by EAex at an early stage, our time-of-addition assay suggested that later stages of virus replication, including viral RNA synthesis, viral protein modification and maturation, virus assembly, and virus release, might also be targets of EAex.

In addition to the direct effect against influenza virus replication, He and Dai reviewed the pharmacological anti-inflammatory effects of BS [29]. Cytokine storm is known to be a major factor determining patients’ conditions [30,31], and treatment aimed specifically at modulating the cytokine storm in order to relieve the severity of patients’ conditions has been demonstrated [32]. Thus, we propose that EAex not only directly inhibits virus replication, but also might play a role in modulating immune responses in our animal model.

## 3. Experimental

### 3.1. Cell Culture and Virus Infection

Madin-Darby canine kidney (MDCK) cells were cultured in E10 containing Dulbecco’s modified Eagle’s medium (DMEM; Invitrogen, Carlsbad, CA, USA), 10% heat-inactivated fetal bovine serum (FBS; JRH Biosciences, Lenexa, KS), 2 mM l-glutamine (Gibco, BRL, Gaithersburg, MD, USA), 0.1 mM nonessential amino acid (NEAA) mixture (Gibco), 100 U/mL penicillin, and 0.1 mg/mL streptomycin (Sigma, Aldrich, Poole, UK). Human rhabdomyosarcoma (RD) cells were cultured in DMEM containing 10% heat-inactivated FBS and penicillin/streptomycin. Human lung carcinoma A549 cells were cultured in minimal essential medium (MEM; Invitrogen, Carlsbad, CA, USA) containing 10% heat-inactivated FBS and penicillin/streptomycin. Influenza virus A/WSN/33 was obtained from the American Type Culture Collection and was propagated in MDCK cells. The sources and proliferation of the other influenza viruses, including H1N1pdm, and enteroviruses have been described previously [33].

### 3.2. Preparation of EA Extract from BS

*Paeonia lactiflora* Pall. was purchased from Sun Ten Pharmaceutical Co., Ltd. of Taiwan in November, 2012. *Paeonia lactiflora* (300 g) were extracted with EtOH (1 L × 2) at 50 °C for 4 h. After evaporation of the solvent *in vacuo*, the concentrated EtOH extract was partitioned between EtOAc and H_2_O to give EtOAc (EAex) and H_2_O portions.

### 3.3. IC_50_ Determination by Anti-CPE (neutralization) Assay

MDCK cells (1.5 × 10^4^ cells/well) were seeded in a 96-well tissue culture plate infected with virus (9TCID_50_, MOI: 1 × 10^−2^ for influenza virus A/WSN/33) in the presence of various concentrations of EAex in E0 (DMEM containing 100 U/mL penicillin, 100 mg/mL streptomycin, 2 mM L-glutamine, 0.1 mM NEAA mixture, and 2.5 mg/mL trypsin). After incubation at 37 °C under 5% CO_2_ for 72 h, the medium was removed and the cells were fixed with 4% paraformaldehyde for 1 h at room temperature, followed by staining with 0.1% crystal violet for 20 min at room temperature. The density of the cells was measured with a microplate reader (VICTOR^3^ Multilabel Reader, Perkin Elmer, Shelton, CT, USA). IC_50_ was defined as the concentration of EAex that could inhibit the virus-induced cytopathic effect (CPE) by 50%. 

### 3.4. IC_50_ Determination by Plaque Reduction Assay

MDCK cells (5 × 10^5^ cells/well) were seeded into six-well tissue culture plates and incubated overnight. The monolayers of cells were incubated with influenza virus at approximately 30–50 PFU/well with or without different concentrations of the compound. After adsorption of the virus for 1 h at 37 °C, the viral suspension was removed, and the cells were washed with PBS. The cells were then overlain with E0 containing 0.3% agarose with indicated compound concentration. After incubation for 48–72 h at 37 °C under 5% CO_2_, the cells were fixed with 4% paraformaldehyde and then stained with 1% crystal violet. The numbers of plaques were counted and the antiviral activity of the compound was calculated with respect to virus only control.

### 3.5. Cytotoxicity Assay and MTT Assay

A 96-well tissue culture plate was seeded with 1.5 × 10^4^ cells/well, which were cultured overnight at 37 °C under 5% CO_2_. The cells were washed once with DPBS before the addition of various concentrations of BS EAex. After incubation at 37 °C for 72 h, the cells were washed once with DPBS and 50 µL/well 3-(4,5-dimethylthiazol-2-yl)-2,5-diphenyltetrazolium bromide (MTT; 0.5 mg/mL) was added, and then the cells were incubated at 37 °C for 3 h. Dimethylsulfoxide (DMSO; 200 µL/well) was added to dissolve the formazan crystals and the optical density of the cells was measured at 570 nm (OD_570_) with a microplate reader. The concentration of EAex that caused the death of 50% of the cells was defined as the 50% cytotoxic concentration (CC_50_).

### 3.6. Time-of-Addition Assay

A six-well tissue culture plate was seeded with MDCK cells (5 × 10^5^ cells/well), which were then incubated at 37 °C for 16–20 h under 5% CO_2_. The cells were infected with influenza virus A/WSN/33 (at the indicated MOIs) at −1 hpi and kept on ice for 1 h. After viral adsorption, the cells were washed with Hank’s balanced salt solution (HBSS) to remove any unbound virus. EAex (0.025 mg/mL) in E0 was added at −3 to −1 h (preadsorption), −1 to 0 h (adsorption), 0 to 12 h, 6 to 12 h, 9 to 12 h, and −3 to 12 h. The supernatants were harvested at 12 hpi and the viral titers were determined by plaque assay.

### 3.7. RNA Extraction and Quantitative Reverse Transcription-PCR (RT-qPCR)

MDCK cells (5 × 10^5^ cells/well) were infected with influenza virus A/WSN/33 (MOI: 0.01) for 1 h and treated with or without EAex. The cells were harvested at 0, 6, 9, and 12 h post-infection (pi) and their total RNA extracted with TRIzol reagent (Invitrogen), according to the manufacturer’s instructions. The total RNA (5 µg) was reverse-transcribed using an M-MLV Reverse Transcriptase Kit (Invitrogen). The resulting cDNA products of reverse transcription were quantified using a Smart Quant Green MasterMix with dUTP and ROX Kit (Protech, Taipei, Taiwan) and detected with a Step One Plus Sequence Detection System (Applied Biosystems, Foster City, CA, USA). The sequences of the primers used for the PCR were: glyceraldehyde phosphate dehydrogenase (GAPDH) forward 5'-AAG AAG GTG GTG AAG CAG GC-3' and GAPDH reverse 5'-TCC ACC ACC CTG TTG CTG TA-3'; matrix protein1 (M1) forward 5'-GAC CAA TCC TGT CAC CTC-3' and M1 reverse 5'-GAT CTC CGT TCC CAT TAA GAG-3'.

### 3.8. Detection of Viral Protein Synthesis and AKT Phosphorylation by Western Blotting

A six-well tissue culture plate was seeded with cells (5 × 10^5^ cells/well), which were then incubated at 37 °C for 16–20 h under 5% CO_2_. The cells were infected with the indicated MOIs of influenza virus A/WSN/33 at 37 °C for 1 h, and then washed twice with HBSS. EAex (0.025 mg/mL) was added and the cells were harvested at the indicated time periods, then washed twice with PBS and lysed with lysis buffer (1% Triton X-100, 0.5% sodium deoxycholate, 150 mM NaCl, 50 mM Tris-HCl (pH 7.5), 2 mM EDTA, and protease inhibitors). The lysates were incubated for 20 min on ice and then centrifuged at 14,000× *g* for 10 min at 4 °C. The supernatants were collected and the protein concentrations were determined using a Bio-Rad Protein Assay Kit (Bio-Rad, Hercules, CA, USA). Equal amounts of proteins were resolved by sodium dodecyl sulfate—(10%) polyacrylamide gel electrophoresis. The proteins were electrotransferred to polyvinylidene difluoride membranes and incubated overnight at 4 °C with primary antibody and then with secondary antibody at 37 °C for 1 h. An AKT phosphorylation assay was performed with A549 cells that had been serum-starved in MEM for 24 h before viral infection. Influenza virus A/WSN/33 (MOI: 0.01) was incubated with 0.025 mg/mL EAex on ice for 1 h. The A549 cells were infected with the EAex-treated influenza virus and harvested after 10, 20, 30, and 60 min. The cell lysates were prepared and subjected to immunoblotting analysis. The viral proteins were visualized with goat anti-influenza virus M1 antibody (ViroStat, Portland, ME, USA). AKT was detected with mouse anti-phospho-AKT (Ser473) antibody (Cell Signaling, Beverly, MA, USA) and rabbit anti-AKT antibody (Santa Cruz Biotechnology, Inc., Santa Cruz, CA, USA). GAPDH was used as an internal control and detected with mouse anti-GAPDH antibody (Santa Cruz Biotechnology). The proteins were detected using an enhanced chemiluminescence western blotting detection system (Millipore, Billerica, MA, USA). Serum treatment was used as a positive control for AKT phosphorylation because serum has been shown to act as a mitogen, and induces a marked increase in AKT phosphorylation [18].

### 3.9. Attachment Assay

A 96-well tissue culture plate was seeded with MDCK cells (2 × 10^4^ cells/well), which were then incubated overnight at 37 °C under 5% CO_2_. The cells were chilled on ice for 20 min and the medium was removed. The cells were infected with 3TCID_50_ of influenza virus A/WSN/33 in the presence of increasing concentrations of EAex on ice for 1 h. The medium containing the unabsorbed virus was removed and the cells were then washed twice with HBSS and maintained in E0. After incubation for 72 h, cell viability was determined with an MTT assay. This attachment method has been described previously [19].

### 3.10. Penetration Assay

A 96-well tissue culture plate was seeded with MDCK cells (2.4 × 10^4^ cells/well), which were then incubated overnight at 37 °C under 5% CO_2_. The cells were chilled on ice for 20 min and the medium was removed. The cells were infected with 3TCID_50_ of influenza virus A/WSN/33 on ice for 30 min. The medium was then removed and the cells were washed twice with HBSS. Serial dilutions of EAex were added and the cells incubated at 37 °C for 1 h to allow the virus to penetrate the cells. The infected cells were then treated with HBSS (pH 2) for 1 min to inactivate any viruses that had not penetrated into the cells, and then HBSS (pH 11) was immediately added to neutralize the buffer. The HBSS was removed, 200 µL of E0 was added, and the cells were incubated at 37 °C for 72 h. An MTT assay was used to measure cell viability.

### 3.11. Hemagglutination Assay (HAv) and Hemagglutination Inhibition (HAI) Assay

Influenza virus A/WSN/33 was serially diluted twofold in PBS and mixed with two volumes of guinea pig red blood cells (RBCs) for 1 h in round-bottomed 96-well plates. The RBC aggregation caused by the lowest viral titer was defined as 1 × HAv. An influenza virus titer of 4 × HAv was used for the HAI assay. In the HAI assay, various concentrations of EAex were mixed with influenza virus A/WSN/33 at room temperature for 30 min. This mixture was then incubated with two volumes of guinea pig RBCs for 1 h.

### 3.12. NA Assay

Influenza virus A/WSN/33 was added to a 96-well tissue culture plate and then mixed with EAex or Relenza (diluted in 32.5 mM MES, 4 mM CaCl_2_, pH 6.5) at 37 °C for 30 min. 20-(4-Methylumbelliferyl)-α-d-*N*-acetyl neuraminic acid (MU-NANA, working concentration of 60 mM) was added as the substrate and incubated at 37 °C for 1 h. The reaction was stopped with the addition of stop solution (0.1 M glycine buffer containing 25% alcohol, pH 10.7). Fluorescence was measured with a microplate reader (VICTOR^3^ Multilabel Reader) at an excitation wavelength of 355 nm and an emission wavelength of 460 nm.

### 3.13. Mouse Experiment

Four-week-old SPF BALB/C mice were purchased from Lasco, Taiwan. All animal protocols were approved by the Institutional Animal Care and Use Committee of the ChangGung University (CGU10-001). EAex was administered orally as indicated, and then the mice were anesthetized prior to nasal challenge with A/WSN/33 (2 × LD_50_, 2 × 10^4^ pfu in 20 µL). EAex was prepared at the indicated concentration in a final volume of 100 µL in 5% DMSO aqueous solution. Body weight was measured every day for 12 days post-challenge. Relenza (100 µg in 100 µL of 5% DMSO aqueous solution) was administered orally twice a day (10 mg/kg/d) as a positive control, and an equal volume of DMSO was used as the negative control.

### 3.14. Indirect Immunofluorescence Assay

Details are provided as Supplementary Materials S1.1.

### 3.15. RNA Polymerase (RdRp) Activity Assay (Minigenome Reporter Assay)

Details are provided as Supplementary Materials S1.2.

## 4. Conclusions

EAex exerts its anti-influenza effects by inhibiting viral entry without producing cytotoxic side effects and, thus, provides a potential agent for antiviral chemotherapeutics.

## Supplementary Files

Supplementary File 1 Supplementary Methods (PDF, 708 KB)
