# Supplementary material for: Characterization of the Anti-Influenza Activity of the Chinese Herbal Plant Paeonia lactiflora
Source: Viruses. 2014 Apr 23;6(4):1861–75. doi: 10.3390/v6041861 (PMC4014724; doi:10.3390/v6041861)

Supplementary Methods

S1.1. Indirect Immunofluorescence Assay

MDCK cells (1×10^5) were seeded onto coverslips and incubated at 37 °C for 16–20 h under 5% CO_2 and then infected or mock infected with influenza virus A/WSN/33 at the indicated MOIs. After the cells were washed with HBSS, EAex medium was added at the indicated time points and the cells were then fixed in 4% paraformaldehyde for 1 h at room temperature. The cells were permeabilized with 0.5% Triton X-100 in PBS for 5 min and blocked with 0.5% bovine serum albumin in PBS for 1 h. The cells were stained with either anti-M1 (ViroStat, Portland, ME, USA), anti-HA antibodies (Abcam, Cambridge, MA, USA), or anti-NP for 1 h and then incubated with the appropriate Alexa-Fluor-488-labeled secondary antibody. The cell nuclei were stained with 4',6-diamidino-2-phenylindole (DAPI). Fluorescence was detected with a Zeiss Axiovert 200 M microscope with a 100× oil-immersion objective lens.

S1.2. RNA Polymerase (RdRp) Activity Assay (Minigenome Reporter Assay)

HEK293 (4×10^4 cells) were seeded in a 48-well plate (precoated with poly-L-lysine for 30 min), then incubated at 37 °C for 16–20 h. Cells were then cotransfected with 0.08 μg of pHW2000-NP, -PA, -PB1, and -PB2, and also with 0.08 μg of pPOLI-FLuc-RT and 0.008 μg of pRL-TK by Lipofectamine 2000 for 6 h. The transfection complex was replaced with HEK293 medium together with the indicated concentrations of EAex. The RNP complex activity was determined using a Dual-Luciferase Assay System (Promega, Madison, WI, USA). Each group contained quadruplicates, and firefly luciferase activity (Fluc) was normalized with Renilla luciferase (Rluc) and the quadruplicate results were then averaged.

Supplementary Results

Figure S1. Inhibition of viral entry in attachment assays. Before infection, MDCK cells were chilled on ice for 20 min. The cells were infected with $3TCID_{50}$ of influenza virus (**a**) A/TW/7717/09 or (**b**) B/99/07, and treated with different concentrations of EAex on ice for 60 min. The medium containing unbound virus was removed, new medium lacking FBS was added, and the cells were incubated for 72 h. Cell viability was measured using an MTT assay. Data are presented as the means ± SD of three independent experiments.

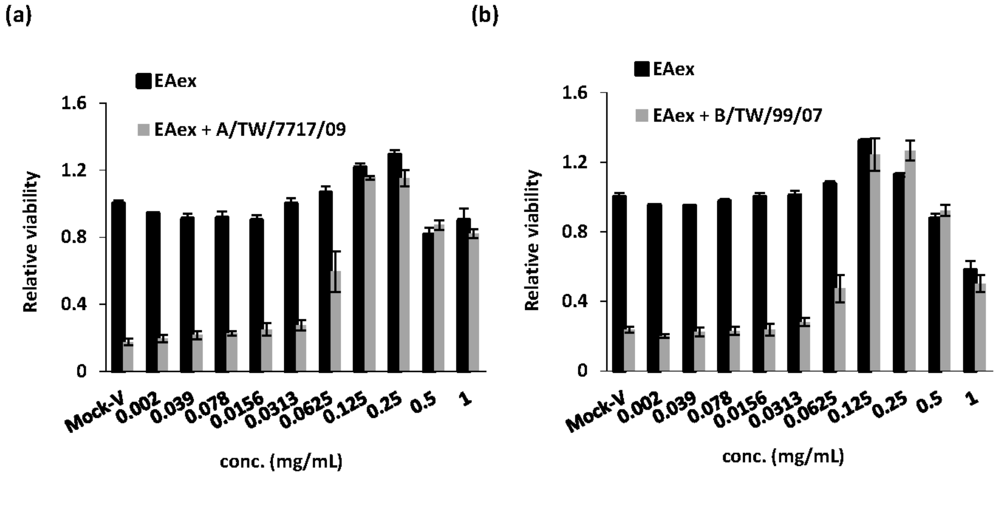

Figure S2. Inhibition of viral entry in penetration assays. MDCK cells were prechilled for 20 min and infected with 3 × $TCID_{50}$ of influenza virus (**a**) A/TW/7717/09 or (**b**) B/99/07 on ice for 30 min. After the unbound virus was removed, the infected cells were supplied with FBS-free medium containing various concentrations of EAex and incubated at 37 °C for 1 h. Finally, the viruses that had not penetrated the cells were inactivated with HBSS (pH 2) and then neutralized with HBSS (pH 11). Cell viability was measured using an MTT assay. Data are presented as the means ± SD of three independent experiments.

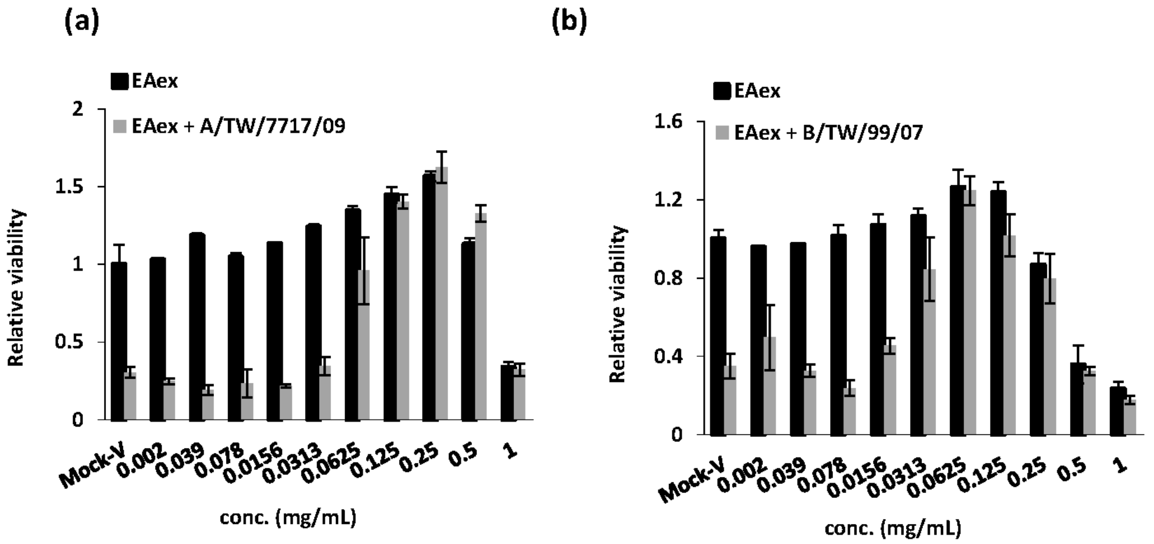

Figure S3. EAex did not inhibit RNP complex activity. HEK293 cells were transfected with PA, PB1, PB2, NP, and Fluc and Rluc plasmids, and then treated with EAex as indicated. The RNP complex activity was determined after (**a**) 24 h or (**b**) 48 h of EAex treatment. The first two groups NP and Fluc were served as negative controls.

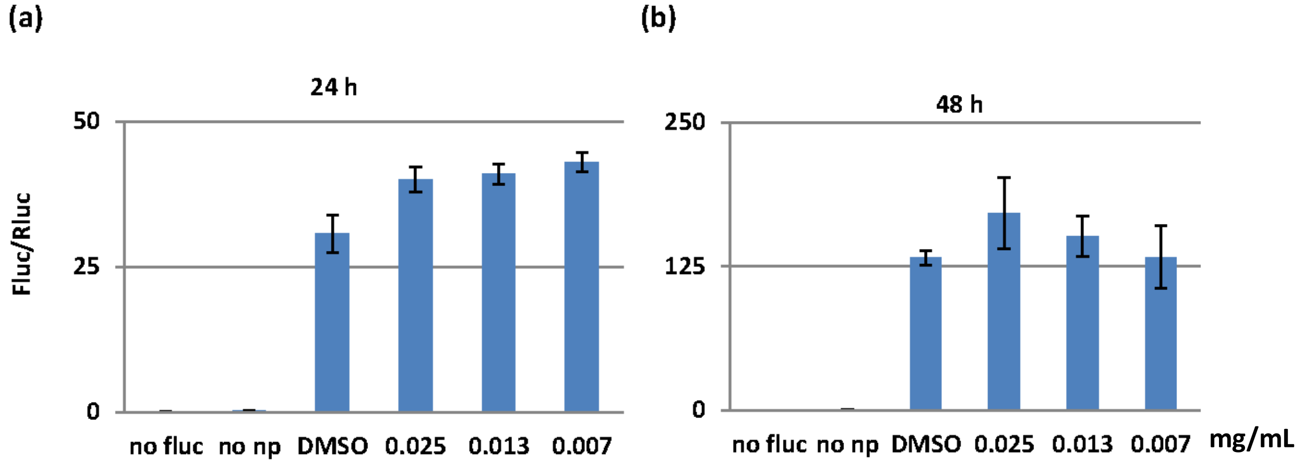

Figure S4. EAex did not alter viral NP, M1, or HA protein cellular localization. MDCK cells were seeded on coverslips overnight before they were infected with influenza virus A/WSN/33 (MOI: 1). After viral adsorption, the infected cells were treated with 0.025 mg/mL EAex and fixed with 4% paraformaldehyde at 6, 9, and 12 h p.i. The green foci (left panel) indicate the presence of viral proteins (**a**) M1, (**b**) NP, and (**c**) HA. The secondary antibodies were Alexa-Fluor-488-conjugated goat anti-mouse immunoglobulin G antibody. The nuclei were stained with DAPI (middle panel). The right panel shows a merged image of the viral protein and DAPI-stained nuclei.

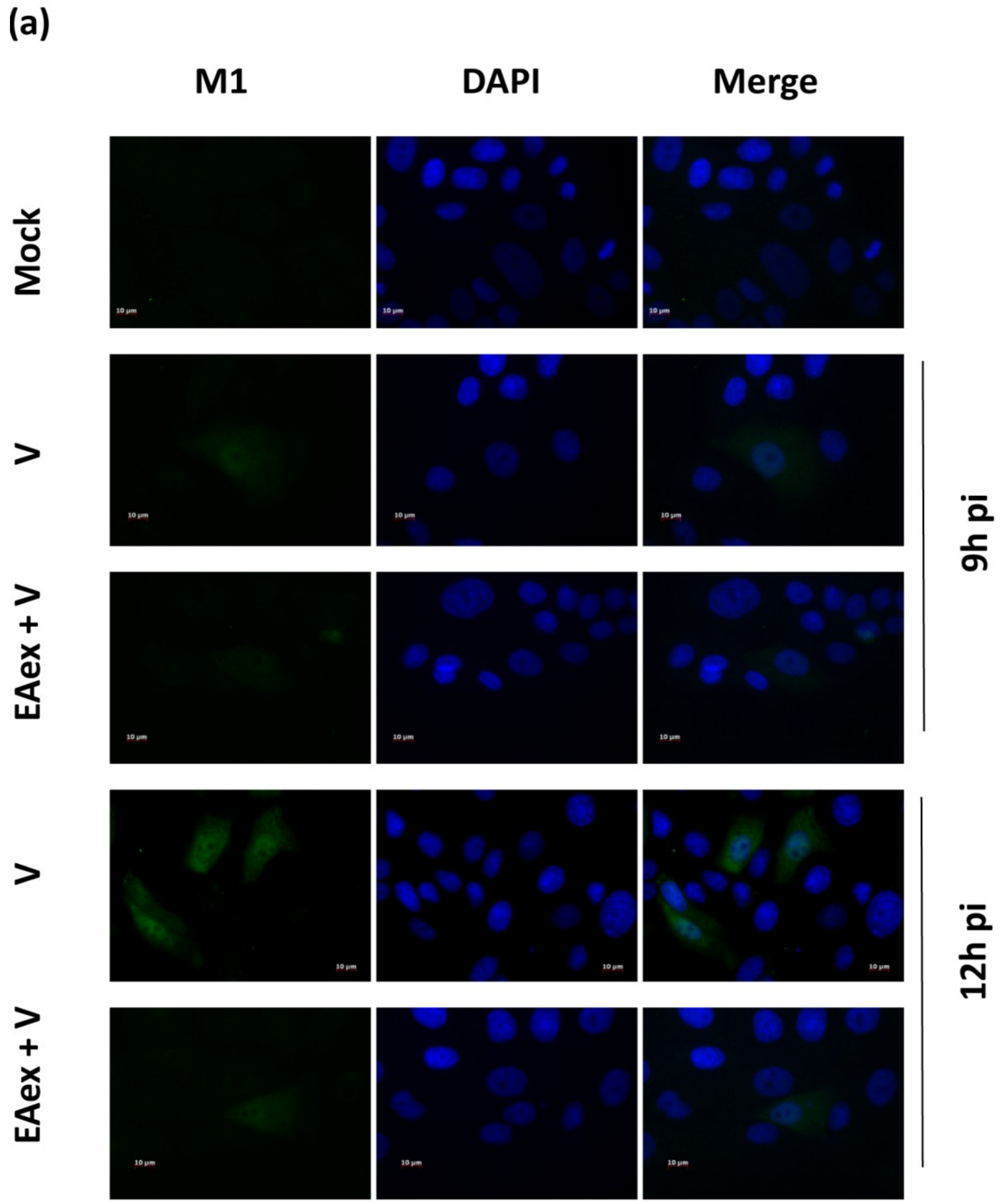

Figure S4. *Cont.*

(b)

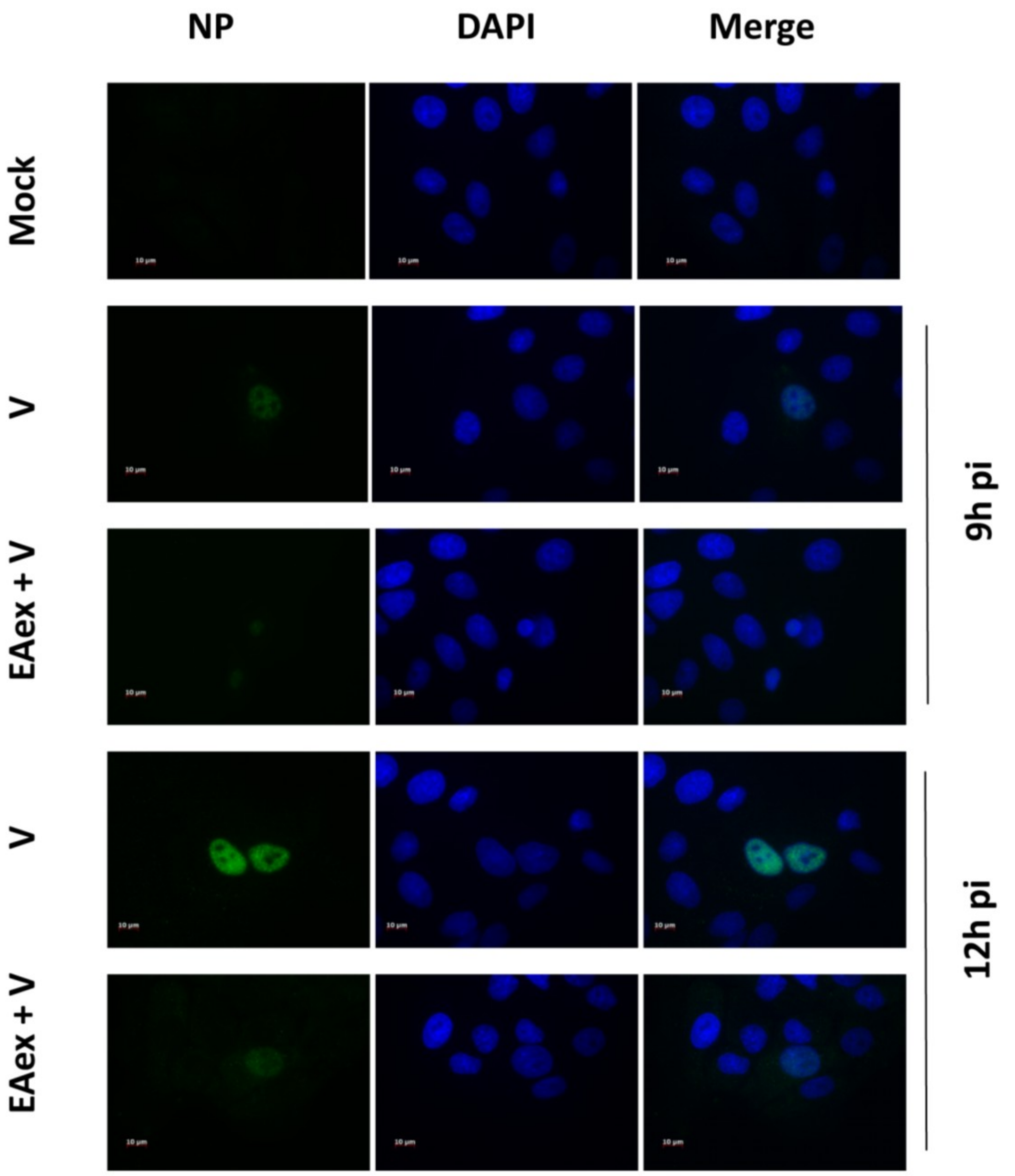

Figure S4. *Cont.*

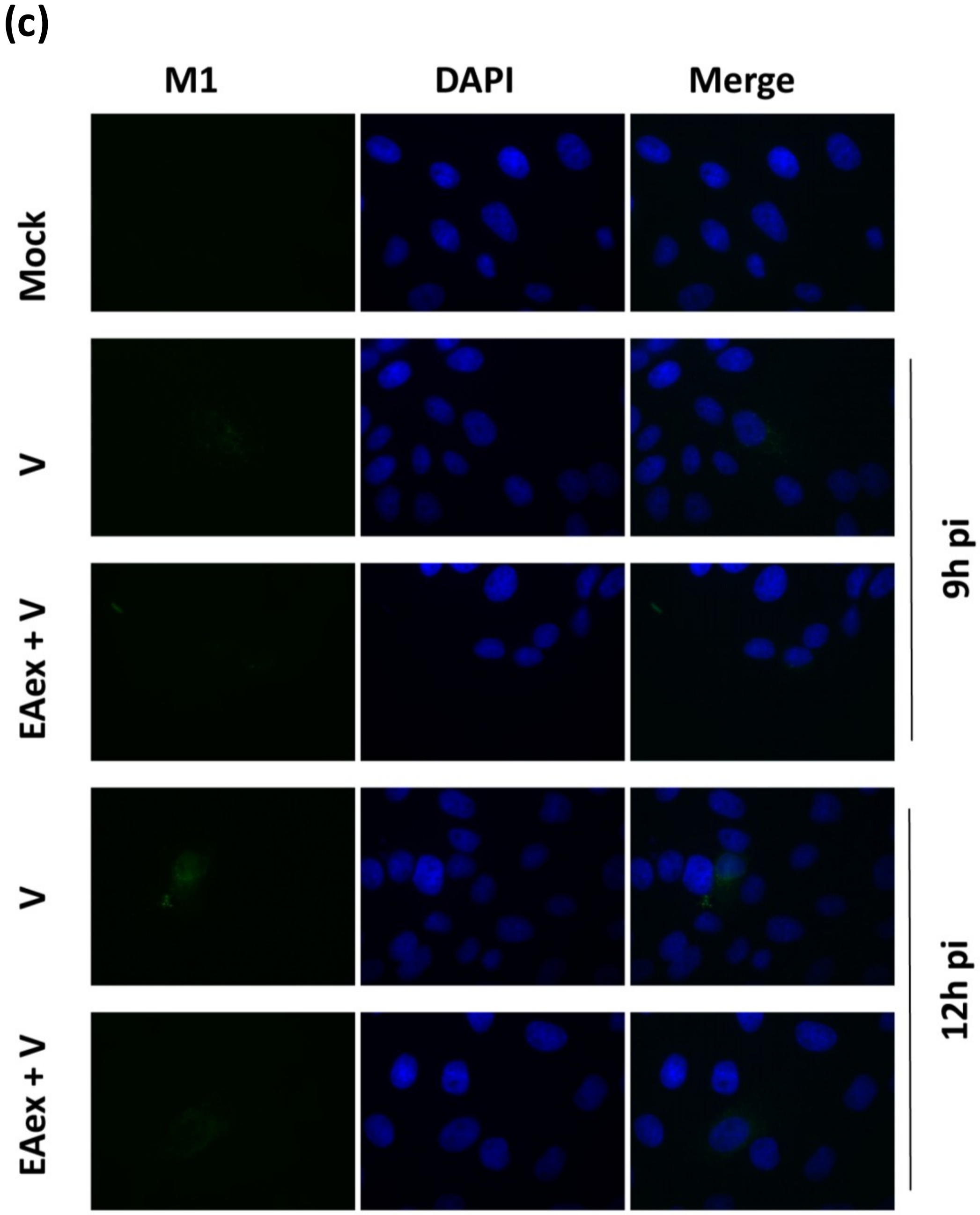

Supplement: Supplementary File 1 — Supplementary Methods (PDF, 708 KB) [file viruses-06-01861-s001.pdf]
